# Mitochondrial genome copy number variation across tissues in mice and humans

**DOI:** 10.1073/pnas.2402291121

**Published:** 2024-08-06

**Authors:** Sneha P. Rath, Rahul Gupta, Ellen Todres, Hong Wang, Alexis A. Jourdain, Kristin G. Ardlie, Sarah E. Calvo, Vamsi K. Mootha

**Affiliations:** ^a^HHMI, Boston, MA 02114; ^b^Department of Molecular Biology, Massachusetts General Hospital, Boston, MA 02114; ^c^Department of Systems Biology and Medicine, Harvard Medical School, Boston, MA 02115; ^d^Broad Institute of Massachusetts Institute of Technology and Harvard, Cambridge, MA 02142; ^e^Department of Immunobiology, University of Lausanne, Epalinges 1066, Switzerland

**Keywords:** mtDNA, TFAM, histone, mitochondrial ribosome, GPX4

## Abstract

The mammalian mitochondrial genome (mtDNA) is multicopy and its copy number (mtCN) varies widely across tissues, in development and in disease. Here, we systematically catalog this variation by assaying mtCN in 52 human tissues across 952 donors (10,499 samples from the Genotype-Tissue Expression project) and 20 murine tissues using qPCR, capturing 50- and 200-fold variation, respectively. We also estimate per cell mtCN across 173 human cell lines from the Cancer Cell Line Encyclopedia using whole-genome sequencing data and observe >50-fold variation. We then leverage the vast amount of genomics data available for these repositories to credential our resource and uncover mtDNA-related biology. Using already existing proteomics data, we show that variation in mtCN can be predicted by variation in TFAM, histone, and mitochondrial ribosome protein abundance. We also integrate mtCN estimates with the CRISPR gene dependency measurements to find that cell lines with high mtCN are resistant to loss of GPX4, a glutathione phospholipid hydroperoxidase. Our resource captures variation in mtCN across mammalian tissues and should be broadly useful to the research community.

The mammalian mitochondrial genome (mtDNA) is polyploid, unlike the nuclear genome which is normally present in two copies in all mononucleated somatic cells. The mtDNA copy number (mtCN) varies >1,000-fold across cell types, ranging from ~100 in blood, to ~7,000 in heart (1), and over 100,000 in murine unfertilized eggs (2). The extent of mtCN variation, however, remains unknown as measurements have been limited to only a few tissues across isolated studies. Broader catalogs of mtCN could serve as valuable resources for understanding mtDNA biology, its age-associated decline, and contributions to disease.

Here, we catalog mtCN in a wide range of human and murine tissues and cell lines for which vast amounts of other multiomic measurements are already available. We illustrate how mtCN can be integrated with these datasets to recover both known and new biology.

## Results and Discussion

**Fig. 1.**
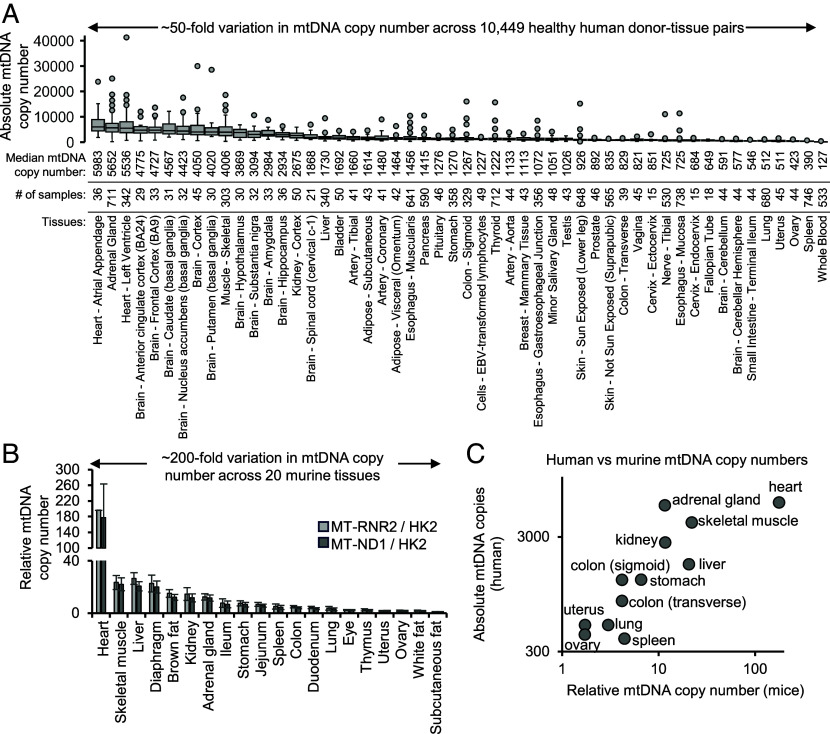
MtDNA copy number variation across human and murine tissues. (*A*) Absolute mtDNA copy number (mtCN) per diploid nuclear genome measured by qPCR in 10,449 human donor-tissue pairs from GTEx. (*B*) mtCN measured by qPCR in 20 murine tissues and normalized to the tissue with the lowest mtCN. Error bars represent SE from biological replicates (n = 3 kidney, n = 4 all other tissues). (*C*) Scatter plot of human and murine mtCNs for tissues common across “*A*” and “*B*.”

We determined absolute mtCN per diploid nuclear genome by qPCR in 10,449 human donor-tissue pairs, representing 52 tissues across 952 donors from the Genotype-Tissue Expression (GTEx) project. We observed ~50-fold variation in median mtCN across tissues and >200-fold interindividual variation in mtCN within a tissue (Fig. 1*A* and Datasets S1–S4). The latter could arise from differences in genetic background, age, disease, and technical factors such as sampling heterogeneity. Our relative ranks match published estimates, for example, 11 human tissues common to our study and Wachsmuth et al. (3) have concordant relative rank order by mtCN.

Next, we measured relative mtCN in 20 tissues of the commonly used C57BL/6J mouse strain by qPCR. We observed ~200-fold variation in mtCN across tissues (Fig. 1*B* and Datasets S1–S4). In tissues where we measured mtCN in both species, the relative order of tissues ranked by mtCN in human vs. mouse is highly concordant (Fig. 1*C*), and as expected, mitochondria-rich tissues such as the heart, muscle, and liver have higher mtCN relative to other tissues. Heart, muscle, liver, kidney, and spleen mtCN also previously showed descending order in mice (4).

**Fig. 2.**
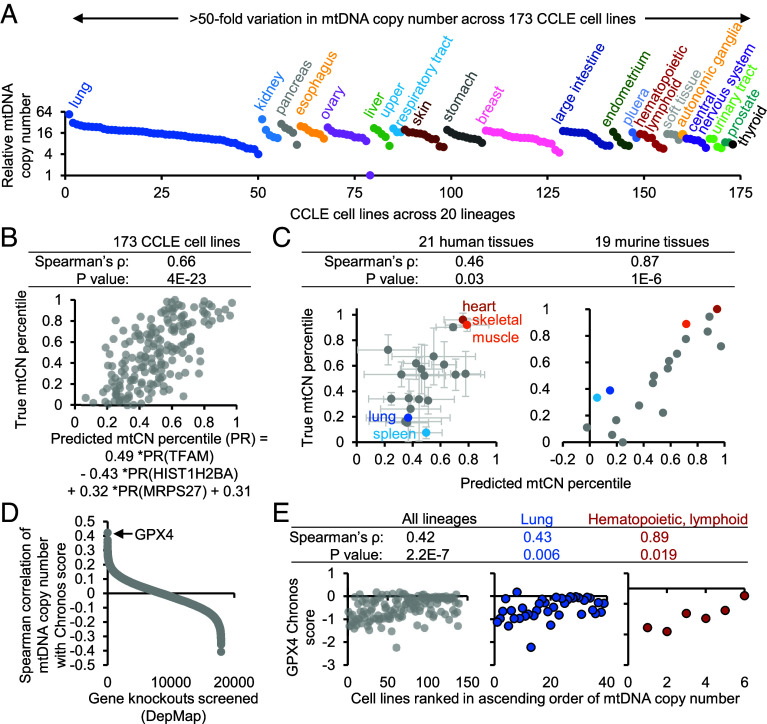
MtDNA copy number analysis across cancer cell lines integrated with protein expression and gene dependency scores. (*A*) mtCN calculated from whole-genome sequencing coverage in 173 cell lines from the CCLE. All estimates are divided by that of the cell line with the lowest mtCN. (*B*) Scatter plot of mtCN calculated in *A* (converted to percentile) vs. mtCN percentiles predicted by a stepwise linear regression model. (*C*) MtCN percentiles were predicted in all 21 human and 19 murine tissues for which protein levels were available. Scatter plots of the measured mtCN from Fig. 1 (converted to percentile) vs. mtCN percentiles predicted by the stepwise linear regression model in (*B*). Error bars represent SD around the mean mtCN percentile across all donors for each tissue. (*D*) Spearman correlation between mtCN and Chronos scores of 17,928 gene knockouts across all 139 CCLE cell lines with joint data. (*E*) Scatter plots of GPX4 Chronos score vs. mtCN rank across lineages and within the two lineages with a significant correlation.

We also calculated mtCN in 173 human cancer cell lines across 20 lineages from the Cancer Cell Line Encyclopedia (CCLE) using whole-genome sequencing coverage (5) of the mitochondrial versus nuclear genome (Fig. 2*A* and Datasets S1–S4). MtCN varies up to 54-fold across all cell lines and up to 23-fold within a lineage (ovary).

We leveraged the joint availability of our mtCN estimates and published tandem mass tag (TMT) proteomics in CCLE (6) to identify proteins whose abundance can explain variation in mtCN. We transformed our mtCN estimates and the TMT data for each of the 5,153 proteins detected in all 173 CCLE cell lines into percentile ranks (PRs) and performed stepwise linear regression without manual preselection of any features, adding parameters until their coefficient’s *P* value exceeded 0.00001 (*SI Appendix*, *Extended Methods*). This yielded a three-parameter model: PR(mtCN) = 0.49*PR(TFAM) − 0.43*PR(HIST1H2BA) + 0.32*PR(MRPS27) + 0.31 (Fig. 2*B*).

The key takeaway from this linear regression is that the abundance of TFAM, histone, and mitochondrial ribosomal protein are predictive of mtCN. The relationship with TFAM is encouraging and well-founded in known biology: This factor is a mitochondrial HMG-box protein that packages mtDNA, is key for its replication, and is known to be limiting for mtCN (7). The inverse relationship with a histone protein makes biological sense as we know that per cell mtCN is highly correlated to cell size (8) but histone concentration anticorrelates with cell size (9). The protein from the small subunit of the mitochondrial ribosome could be predictive of mtCN because more mtDNA could lead to higher mt-rRNA expression, which stabilizes the mitochondrial ribosome and limits the degradation of its protein subunits (10).

Although we trained our model on cancer cell lines, it generalizes in vivo and explains some of the variation in mtCN across human and murine tissues in Fig. 1. In the subset of 94 donor-tissue pairs in GTEx for which mtCN and proteomic measurements of TFAM, HIST1H2BA, and MRPS27 are available (11), our model’s predictions are significantly correlated with the observed mtCN percentile (Spearman ρ = 0.46, *P* = 0.03; Fig. 2 *C*, *Left*). We also applied our model to predict mtCN percentiles of murine tissues using published SILAC proteomics (12). Although HIST1H2BA and MRPS27 were not captured and hence replaced in the model with representative and highly correlated family members (HIST1H2BM and MRPS23), the model still robustly predicted mtCN percentiles that were highly concordant with our measured estimates (Spearman ρ = 0.87, *P* = 1e-6; Fig. 2 *C*, *Right*).

We also correlated our mtCN estimates with genome-wide CRISPR dependency Chronos scores (higher scores indicate tolerance to gene knockout) across CCLE for each of 17,928 gene knockouts (13). We found that high mtCN correlated most strongly with tolerance to loss of GPX4 (rank #1, Fig. 2*D* and Datasets S1–S4). This correlation is observed across all 139 cell lines with joint mtCN and gene dependency data, is particularly strong in the hematopoietic/lymphoid lineage, and is also preserved in the lung lineage, for which joint data were available for the most cell lines (Fig. 2*E*). Consistent with GPX4 being a selenoprotein and an essential output of selenocysteine metabolism (14), “Selenocysteine Synthesis” scored as the most significant pathway in enrichment analysis of the top 100 genes whose loss is tolerated in cells with high mtCN (Datasets S1–S4). GPX4 is a mitochondrial and cytosolic glutathione phospholipid hydroperoxidase commonly linked to protection against lipid peroxidation and ferroptosis. We previously showed that GPX4 loss impairs cell fitness in the face of mtDNA depletion (15).

Currently, the mechanisms underlying variation in mtCN across organs, why mtCN declines in aging, and how it contributes to disease is unknown. Integrating our catalogs of mtCN with other multiomic measurements and chemical-genetic screens may provide insight into these important questions.

## Methods

Human and murine mtCNs were determined by qPCR using the ratio of a mitochondrial vs nuclear DNA amplicon. Mean mtCN for CCLE cell lines was calculated from whole-genome sequencing (5) as (mitochondrial genome coverage) × 2/(nuclear genome coverage). Stepwise linear regression was performed using mtCN percentile ranks (dependent variable) and percentile rank-transformed levels of all 5,153 proteins detected across all 173 cell lines (6) (independent variables). Coefficients were added until their *P* value > 0.00001, yielding a model with three proteins (F-statistic = 44.85, 170 degrees of freedom, *P* = 2.2e-16). To predict mtCN percentiles in human and murine tissues using this model, published TMT (11) and SILAC proteomics (12) were used, respectively. See *SI Appendix* for detailed methods.

## Supplementary Material

Appendix 01 (PDF)

Dataset S01 (XLSX)

## Data Availability

Data plotted in the figures is in Datasets S1–S4. Code for the stepwise linear regression is deposited here: https://github.com/MoothaLab/2024_mtCN_stepwise_regression (16). All other data are included in the manuscript and/or supporting information.
