# Supplementary material for: Mitochondrial genome copy number variation across tissues in mice and humans
Source: Proc Natl Acad Sci U S A. 2024 Aug 6;121(33):e2402291121. doi: 10.1073/pnas.2402291121 (PMC11331085; doi:10.1073/pnas.2402291121)
Supplement: Supplementary file 1 — Appendix 01 (PDF) [file pnas.2402291121.sapp.pdf]

PNAS

Supporting Information for
Mitochondrial genome copy number variation across tissues in mice and humans

Sneha P. Rath[a,b,c,d], Rahul Gupta [a,b,c,d], Ellen Todres[d], Hong Wang [a,b,c,d], Alexis A. Jourdain [a,b,c,d,e], Kristin G. Ardlie[d], Sarah E. Calvo[a,b,c,d,1], Vamsi K. Mootha [a,b,c,d,1]

[a] Howard Hughes Medical Institute
[b] Department of Molecular Biology, Massachusetts General Hospital, Boston, MA, USA.
[c] Department of Systems Biology and Medicine, Harvard Medical School, Boston, MA, USA.
[d] Broad Institute of MIT and Harvard, Cambridge, MA, USA.
[e] Department of Immunobiology, University of Lausanne, Epalinges, Switzerland.
[1] Corresponding authors: Vamsi K. Mootha and Sarah E. Calvo
Email: vamsi_mootha@hms.harvard.edu and scalvo@broadinstitute.org

This PDF file includes:

Extended Methods
SI References
Supplementary dataset descriptions

Other supporting materials for this manuscript include the following:

Supplementary datasets (Excel sheet, four tabs)

Extended Methods

Human mtDNA copy numbers across tissues from GTEx:
Post-mortem human tissue samples from the GTEx consortium were cut in half, with one piece used for DNA extraction (via PAXgene) and the other for RNA extraction (via PAXgene) as described by the GTEx consortium [1]. DNA samples were plated on 96-well plates, and all plates included two control samples which were shared between all plates. All extracted DNA samples were diluted to a concentration of 0.4 µg/µL. For quantification, we generated qPCR primers (IDT) as well as probes for a multiplexed TaqMan assay (Applied Biosciences). Primers were diluted in ultra-pure water to 100 µM, and probes were at 100 µM in TE. One set of probes and primers targeted a sequence within ND2 on the mitochondrial DNA and another targeted B2M on the nuclear genome. The primers were as follows:
ND2-Forward: tgttggttatacccttcccgtacta
ND2-Reverse: cctgcaaagatggtagagtagatga
ND2-Probe: 6FAM-ccctggcccaaccc-MGBNFQ
B2M-Forward: GCTGGGTAGCTCTAAACAATGTATTCA
B2M-Reverse: CCATGTACTAACAAATGTCTAAAATGGT
B2M-Probe: VIC-CAGCAGCCTATTCTGC-MGBNFQ
Probes were combined in a stock solution, comprising 50 µL of each of the probes, 10 µL of each of the ND2 primers, 250 µL of each of the B2M primers, and 380 µL of water. 1 µL of the probe mix was added to 10 µL of the Taqman master mix, 8 µL of water, and 1 µL of DNA for each reaction. The qPCR protocol was as follows, imaging all colors:

1. 95 °C, 10 min
2. 95 °C, 15 sec
3. 55 °C, 15 sec
4. 60 °C, 1 min
5. Picture
6. Cycle to 2, 40x

Batch effects due to usage of a different TaqMan master mix lot were initially observed by tracking quantification of the control samples from run-to-run. We determined the average change in ND2 and B2M Ct from batch 1 to batch 2 across a set of the same samples; this constant transformation was then applied to all data obtained using the affected lot and was sufficient to alleviate any observed batch effects.

We constructed calibration curves to convert qPCR readings to mtCN estimates. To do so, we generated synthetic plasmids at known concentration containing segments of the ND2 and B2M genes respectively which contained target sequences for the above primers and probes cloned into pUC-Kan plasmids. Serial dilutions were performed for each plasmid ranging from 1 ngDNA/µL to 1e-7 ngDNA/uL and the aforementioned TaqMan qPCR protocol was used to estimate Ct values at each known concentration. Six replicates of the standard curve were performed across 2 experiments. Estimates were averaged within each experiment; the subsequent per-experiment average Ct values were then averaged once again to produce final Ct estimates for each serial dilution of each plasmid. These data were used to fit linear models of the form logConcentration ~b0 + b1*Ct for ND2 and B2M respectively. For each assayed human sample, these models were used to convert measured Ct values for ND2 and B2M into estimates of mtDNA (via ND2) and nucDNA (via B2M) concentration. mtCN was estimated as [ND2]*2/[B2M].

The specific sequences cloned into the plasmids are below, with sites of forward and reverse primers and internal probes highlighted in yellow, cyan, and green respectively:

pUC-Kan-ND2
ATATGTCTGATAAAAGAGTTACTTTGATAGAGTAAATAATAGGAGCTTAAACCCCCTTATTTCT
AGGACTATGAGAATCGAACCCATCCCTGAGAATCCAAAATTCTCCGTGCCACCTATCACACC
CCATCCTAAAGTAAGGTCAGCTAAATAAGCTATCGGGCCCATACCCCGAAAATGTTGGTTAT
ACCCTTCCCGTACTAATTAATCCCCTGGCCCAACCCGTCATCTACTCTACCATCTTTGCAGG

CACACTCATCACAGCGCTAAGCTCGCACTGATTTTTTACCTGAGTAGGCCTAGAAATAAACAT
GCTAGCTTTTATTCCAGTTCTAACCAAAAAAATAAACCCTCGTTCCACAGAAGCTGCCATCAA
GTATTTCCTCACGCAAGCAACCGCATCCATAATCCTTCTAATAGCTATCC

pUC-Kan-B2M
TCCTCTAGCTTTTGTGGCAGCTTCAGGTATATTTAGCACTGAACGAACATCTCAAGAAGGTAT
AGGCCTTTGTTTGTAAGTCCTGCTGTCCTAGCATCCTATAATCCTGGACTTCTCCAGTACTTT
CTGGCTGGATTGGTATCTGAGGCTAGTAGGAAGGGCTTGTTCCTGCTGGGTAGCTCTAAAC
AATGTATTCATGGGTAGGAACAGCAGCCTATTCTGCCAGCCTTATTTCTAACCATTTTAGACA
TTTGTTAGTACATGGTATTTTAAAAGTAAAACTTAATGTCTTCCTTTTTTTTCTCCACTGTCTTT
TTCATAGATCGAGACATGTAAGCAGCATCATGGAGGTAAGTTTTTGACCTTGAGAAAATGTTT
TTGTTTCACTGTCCTGAGGACTATTTATAGACAGCTCTAACATGATAA

Murine mtDNA copy numbers across tissues: Four 8-10-week-old female C57BL/6J mice were euthanized with carbon dioxide and their whole organs were harvested (n=3 for kidney, n=4 for all other organs), quickly washed in PBS, and flash frozen in liquid nitrogen. Total genomic DNA was extracted using the QIAamp DNA micro kit (Qiagen Cat. No. 56304) following manufacturer's protocol and 30 ng DNA was used for each qPCR reaction. Two mitochondrial probes were each normalized to a nuclear probe to calculate mtDNA copy number relative to nuclear genome copies.
Sequences of qPCR primers (forward/reverse):
Mouse_MT-16S: CCGCAAGGGAAAGATGAAAGAC / TCGTTTGGTTTCGGGGTTTC
Mouse_MT-ND1: CTAGCAGAAACAAACCGGGC / CCGGCTGCGTATTCTACGTT
Mouse_nuclear HK2: GCCAGCCTCTCCTGATTTTAGTGT /
GGGAACACAAAAGACCTCTTCTGG

CCLE mtDNA copy numbers: Mean sequence coverage of mtDNA and nuclear DNA was calculated from available whole genome sequence data [2] using Samtools idxstats. Mean mtDNA copy number per cell was estimated by: (mitochondrial genome coverage) x 2 / (nuclear genome coverage).

Linear regression: Both WGS and TMT proteomics data are available for 173 CCLE cell lines. mtDNA copy number was calculated based on the WGS data for each cell line as described above and converted to percentile ranks (rank/173). Next, the same percentile rank transformation was applied to the data for each of 5,153 proteins measured by TMT proteomics [3] across all 173 cell lines. We performed stepwise linear regression with the mtCN percentile rank as the dependent variable, and percentile ranks for all 5,153 proteins as the independent variables. We did not pre-select any features manually. Stepwise regression was performed using regsubsets(nvmax=3, method="forward") from the R package "leaps" (https://CRAN.R-project.org/package=leaps). We used a tolerance level of 0.00001 (parameters added so long as t-statistic p value < 0.00001) to keep the model simple and interpretable. This resulted in a final model with three protein parameters (F-statistic=44.85 with 170 degrees of freedom and p=2.2e-16).

To predict mtDNA copy number percentiles across murine and human tissues using this model, published datasets of TMT proteomics of human tissues in GTEx [4] and SILAC proteomics of murine tissues [5] were used. While all three model parameters were detected in the GTEx proteomics, HIST1H2BA and MRPS27 were not detected in the murine proteomics – for this reason we used another representative and highly correlated protein family member (HIST1H2BM and MRPS23 respectively) that was detected.

SI References

1. GTEx Consortium. Human genomics. The Genotype-Tissue Expression (GTEx) pilot analysis: multitissue gene regulation in humans. Science. 2015 May 8;348(6235):648-60.
2. M Ghandi et al., Next-generation characterization of the Cancer Cell Line Encyclopedia. *Nature.* **569**(7757):503-508 (2019).

3. DP Nusinow et al., Quantitative Proteomics of the Cancer Cell Line Encyclopedia. *Cell.* **180**(2):387-402.e16 (2020).
4. L Jiang et al., A Quantitative Proteome Map of the Human Body. *Cell.* **183**(1):269-283.e19 (2020).
5. T Geiger et al., Initial quantitative proteomic map of 28 mouse tissues using the SILAC mouse. *Mol Cell Proteomics.* **12**(6):1709-22 (2013).

Supplementary Dataset S1 (separate Excel sheet, tab 1): mtDNA copy numbers for human tissues. MtCN for individual donor-tissue pairs from the Genotype-Tissue Expression project (GTEx) and summary statistics corresponding to Fig. 1A

Supplementary Dataset S2 (separate Excel sheet, tab 2): mtDNA copy numbers for murine tissues, data corresponding to Fig. 1B

Supplementary Dataset S3 (separate Excel sheet, tab 3): mtDNA copy numbers for human cancer cell lines from the Cancer Cell Encyclopedia (CCLE), data corresponding to Fig. 2A

Supplementary Dataset S4 (separate Excel sheet, tab 4): Correlation of mtDNA copy number with Chronos score (tolerance to gene losses, DepMap), data corresponding to Fig. 2D and output from geneset enrichment analysis using Enrichr.
